# A Combined Approach of High-Frequency rTMS and Food-Inhibition Association Training Reduces Chocolate Snack Consumption

**DOI:** 10.3389/fpsyt.2019.00815

**Published:** 2019-11-15

**Authors:** Hyeon Min Ahn, Byung-Joo Ham, Sang Hee Kim

**Affiliations:** ^1^Department of Brain and Cognitive Engineering, College of Medicine, Korea University, Seoul, South Korea; ^2^Department of Psychiatry, College of Medicine, Korea University, Seoul, South Korea

**Keywords:** food intake, chocolate, go/no-go task, rTMS, DLPFC

## Abstract

The ability to control impulsive urges is important for maintaining healthy eating habits. Various training strategies have been developed to reduce impulsive urges for food and strengthen cognitive control over tempting food intake. One frequent strategy uses food-inhibition association to alter the associative process between food cues and impulsive urges. Another strategy, repetitive transcranial magnetic stimulation (rTMS) over the dorsolateral prefrontal cortex (DLPFC) to strengthen cognitive control, has received increased attention. Findings, so far, are mixed and limited due to small effect size, interpretational ambiguity, and lack of standardized brain stimulation parameters. We examined whether tempting chocolate snack intake is modulated by food-inhibition association training combined with high-frequency rTMS. In Experiment 1, healthy young adult female volunteers [body mass index (BMI) range, 17–27] performed a food go/no-go task in which chocolate images were consistently paired with either a no-go cue (no-go group, n = 14) or a go cue (go group, n = 14), or both go and no-go cues at equal frequencies (neutral group, n = 15). In Experiment 2, we examined the effect of combined treatment with high-frequency rTMS and food go/no-go training. Sixty healthy young adult female volunteers (BMI range, 15–31) were randomly assigned to one of four groups with equal numbers of participants: rTMS/no-go, rTMS/neutral, sham/no-go, or sham/neutral. rTMS or sham stimulation was applied over the left DLPFC prior to the food go/no-go training task. After training, in both experiments, a taste test was conducted, and the amount of snack intake was measured. In Experiment 1, the no-go training group consumed fewer chocolate snacks than the go training group. No difference was found between the no-go and neutral training groups. In Experiment 2, combined rTMS and no-go training effectively reduced chocolate snack intake compared with neutral training. Although limited by the small sample size, our results suggest the therapeutic potential of combined high-frequency rTMS and food-inhibition association training in enhancing control over the intake of tempting foods in individuals with overeating.

## Introduction

As modern social and environmental changes increase individuals’ exposure to high-calorie, energy-dense food, the imbalance between calorie intake and expenditure continues to grow and contribute to the increased rate of overweight and obesity (1). Because high-calorie, palatable foods typically contain intrinsically rewarding ingredients, such as sugar and fat, and tend to elicit a compelling urge to consume (2), one’s ability to control impulsive urges is important in maintaining healthy eating in everyday life.

According to dual-process models (3), overeating can be explained by overly strong impulsive temptations for food developed by previous association with rewarding experiences and/or relatively weak control over these maladaptive temptations (4). Among various training techniques that aim to alter the associative processes in response to food cues, food-inhibition association training is one of the most widely used methods (2, 5, 6). During the training, food images are repetitively and consistently paired with a cue to inhibit explicit responses. Over the trials, participants gradually develop automatic associations between food images and the engagement of inhibitory responses, which may result in reduced temptation for foods. Various outcome measures, such as food intake, food choice, and self-rated craving, have been tested following such training (6, 7). Overall, the training effect was associated with small to moderate effect sizes in individuals with normal weight and overweight (6). However, ambiguity remains regarding the interpretation of the effect; in some studies, the positive effects of food no-go training were identified only in comparison with go training in which food images are consistently associated with go cues. Therefore, it is unclear whether the effects were due to induced changes associated with no-go training or partly associated with go training (6). The current study attempted to examine the specific effect of no-go training in heathy females with normal weight and also examine whether its effect is improved when used in combination with high-frequency repetitive transcranial magnetic stimulation (rTMS).

Increased attention has been directed toward the use of noninvasive brain stimulation techniques such as rTMS in increasing inhibitory control over the urges for tempting high-calorie foods. In a previous study involving healthy female participants, a single session of high-frequency rTMS or sham stimulation was applied over the left dorsolateral prefrontal cortex (DLPFC) and self-reported cravings were assessed in response to pictures of appetitive snack foods before and after rTMS stimulation (8). Results showed a relative decrease in cravings after rTMS compared with sham stimulation. However, actual food consumption assessed with a taste test did not differ between the rTMS and sham groups (8). Recent meta-analytic studies in healthy adults and patients with eating disorders have found that single session brain stimulation over the left DLPFC is moderately effective in reducing food cravings but its effects on actual food consumption are less pronounced (9, 10). The mixed and relatively small effect of high-frequency brain stimulation might be explained by nonspecific regional activation by rTMS. The DLPFC, the most frequent target of brain stimulation, has been implicated in various high-level cognitive processes such as working memory, planning, and self-control (11–14). Given the complexity of the neural networks associated with the DLPFC, high-frequency rTMS may activate not only the target process critical for controlling urges but also other unrelated cognitive processes. Therefore, brain stimulation is expected to be more effective when paired with a cognitive task that can activate specific target circuits so that the stimulation mostly influences those active circuits and related cognitive processes (15–17). From this point of view, it would be optimal to combine high-frequency rTMS with cognitive training to efficiently intervene in the cognitive mechanisms underlying problematic overeating.

The current study was designed to examine the effect of food-inhibition association training on the consumption of tempting foods. We conducted a series of two experiments with different groups of healthy participants. In the first experiment, we employed a food go/no-go task for food-inhibition association training and examined its effects on food consumption. In the second experiment, we employed a combination of food go/no-go training and high-frequency rTMS over the left DLPFC and examined whether the combined treatment provided greater benefit than food-inhibition training alone. The left DLPFC was selected as the target of stimulation because previous brain stimulation studies revealed that performance on a go/no-go task was dependent on facilitatory and inhibitory brain stimulation of the left DLPFC (18, 19).

For both experiments, we recruited healthy young adult females who regularly consumed snacks. In Experiment 1, the participants were assigned to 1 of 3 groups: go, no-go, or neutral training. The go group was presented with pictures of chocolate that were always associated with go responses, and the no-go group was presented with pictures of chocolate that were always associated with no-go responses (i.e., response inhibition). The neutral group was presented with pictures of chocolate that were associated with either go or no-go responses at equal frequencies. All participants subsequently completed a taste test in which they rated their taste preference for three kinds of chocolate snacks. We tested whether the no-go group would consume fewer chocolate snacks than the other groups. In Experiment 2, participants were randomly assigned to one of four groups: rTMS/no-go, rTMS/neutral, sham/no-go, or sham/neutral. No-go and neutral training were as described for Experiment 1. Experiment 2 did not have go training groups because our primary interest was to identify the effect of no-go training relative to neutral training. For the rTMS groups, prior to the no-go/neutral training, high-frequency rTMS was applied to the left DLPFC. We predicted that the group that underwent combined rTMS/food no-go training would have a greater reduction of chocolate consumption compared with the other groups.

## Materials and Methods

### Experiment 1

*Participants.* A total of 43 healthy female college students [mean age = 21.51 years, SD = 2.63; body mass index (BMI) range = 17.16–26.57 kg/m^2^] who reported being consumers of snacks were recruited from the same academic institution. All participants provided written informed consent. Experimental procedures were approved by the Institutional Review Board of the institution and adhered to the Declaration of Helsinki. The participants were monetarily compensated for their time. Participants were randomly allocated to 1 of 3 groups: no-go (n = 14), neutral (n = 14), or go (n = 15). One participant from the no-go group was excluded from data analysis because she correctly guessed the experimental hypothesis on the role of go/no-go training on chocolate snack consumption during debriefing. **Table 1** shows participant characteristics across the different groups. Participants’ preference for snacks was assessed using a single question on a four-point Likert scale (1: like very little, 4: like very much) and did not differ significantly between groups (**Table 1**). As indicated in **Table 1**, participants in each group were similar in age, BMI, and level of subjective hunger reported prior to the taste test. Furthermore, self-reported impulsiveness as assessed by the Barratt Impulsiveness Scale (BIS-11) (20), intention to restrict food intake as assessed by the Restraint Scale (RS) (21), and mood scores as assessed by the Positive and Negative Affect Scale (PANAS) (22) did not differ across groups.

**Table 1:** Participant characteristics by group in Experiment 1. Means and standard deviations are shown. Statistical results from one-way ANOVAs with a factor of group are presented.

	No-Go	Neutral	Go	*F*(2,39)	*p*
Age	21.54 (2.90)	22.21 (3.38)	20.80 (1.37)	1.03	0.37
BMI	20.74 (2.50)	19.68 (1.77)	20.47 (1.93)	<1	0.39
Hunger	2.00 (0.41)	2.21 (0.70)	2.40 (0.51)	1.82	0.18
Snack preference (1–4)	3.08 (0.76)	3.14 (0.77)	2.93 (0.46)	<1	0.69
Impulsivity (BIS-11)	50.00 (8.25)	47.57 (10.18)	47.60 (11.81)	<1	0.78
PANAS—Positive affect	24.69 (7.58)	24.86 (5.49)	23.60 (6.70)	<1	0.86
PANAS—Negative affect	13.31(3.40)	14.07 (3.25)	13.93 (4.01)	<1	0.84
Restraint scale (RS)	13.92 (5.07)	15.43 (4.83)	13.53 (5.22)	<1	0.58

F is the F-statistics from one-way ANOVA; P is the probability value from one-way ANOVA.

**Figure 1:**
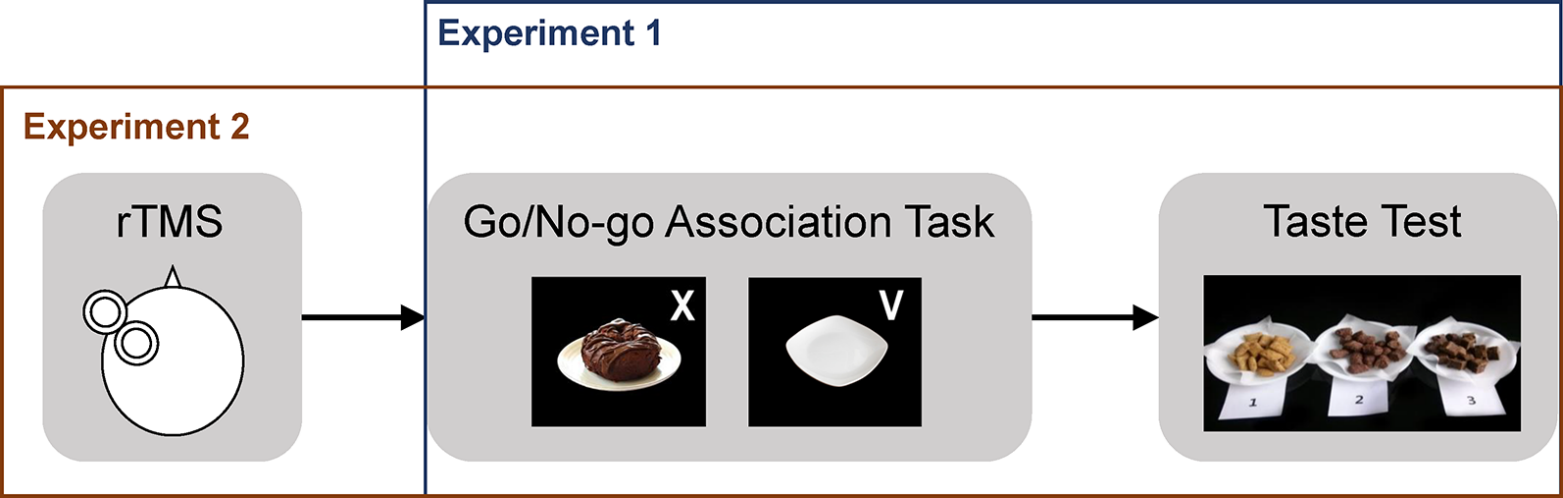
Illustration of the Experiment 1 and 2 procedures.

*Food-inhibition association training.* A food go/no-go task was used for inhibitory association training. It was administered using E-Prime 2.0 (Psychology Software Tools, Inc. Pittsburgh, PA). The go/no-go task consisted of three blocks of 160 trials (480 trials total). Each task trial began with a fixation point that appeared on the screen for a variable length of time (300, 400, 500, or 700 ms). Next, participants were presented with a picture on the center of the computer screen accompanied by either a go or a no-go cue (approximately 1500 ms). The go and no-go cues were letters and were displayed randomly in one of four corners of the picture (**Figure 1**). Picture stimuli consisted of three sets: 10 pictures of various chocolate snacks (e.g., brownies, chocolate cookies, and chocolate donuts), 10 pictures of empty plates, and 20 filler pictures of various healthy foods (e.g., salad, tofu, and steamed vegetables). Pictures were associated with either a go or a no-go cue at equal frequencies.

The association of chocolate snacks and cues differed systematically across different groups. For the no-go group, pictures of chocolate snacks were always paired with the no-go cue, while the pictures of empty plates were paired consistently with the go cue. For the go group, pictures of chocolate snacks were always paired with the go cue, while the pictures of empty plates were paired consistently with the no-go cue. For the neutral group, pictures of chocolate snacks were paired with either the go or no-go cue at equal frequencies. For all groups, pictures of healthy foods were associated with the go or no-go cue at equal frequencies.

*Taste test.* Participants’ actual consumption of chocolate snacks was measured using an unobtrusive taste test (23). In this test, three types of chocolate snacks were prepared in small, similarly sized pieces and served on separate plates, each marked with a number between 1 and 3. The plates contained chocolate chips (20 pieces, 1.55 kcal/piece), cookies with chocolate filling (20 pieces, 8.19 kcal/piece), or brownies (18 pieces, 11 kcal/piece). An in-house version of the taste perception rating sheet, containing 10 items, was used for participants to rate the taste, texture, smell, and preference of each individual snack using seven-point scales ranging from “not at all” to “extremely.” In the test, participants were instructed to eat at least one piece of each snack type to perform the taste ratings, but they were also informed to consume as many of the snacks as they liked because the remaining snacks would be discarded. Participants were left alone for 10 min for this task. Participants’ snack consumption was calculated as the number of kilocalories consumed. The consumed kilocalories for each individual snack were added to determine the total consumption for each participant.

*Procedures.* Upon arrival, participants were informed that that they would be participating in two separate studies: a response inhibition task and a taste perception task. After giving consent, participants received instructions for the go/no-go task. They were instructed to press the space bar as quickly as possible in response to a go cue in the picture but to refrain from pressing the space bar in response to a no-go cue. After completion of the go/no-go task, participants reported their current level of subjective hunger on a three-point scale (1: not hungry at all, 2: hungry, 3: very hungry).

Next, participants performed the taste test and evaluated the taste of chocolate snacks for 10 min. After the taste test, participants were asked to report if they thought that the two studies were related and/or if the first study influenced their performance in the second study. Participants were debriefed and thanked for their participation.

### Experiment 2

*Participants.* The participants were 60 healthy female college students (mean age = 22.55 years, SD = 2.7; BMI range = 14.88–30.82 kg/m^2^) who reported being consumers of snacks. The participants had no current or past history of eating disorders. According to the self-report survey of the amount of money spent on snacks, 40% of participants paid approximately $5–10, 55% paid approximately $1–5, and 5% paid approximately $1 every week to buy snacks. Exclusion criteria included pregnancy, current and past history of psychiatric disorders or neurological illnesses, use of any psychotropic medications, and the presence of implanted metal devices (e.g., pacemakers and metal braces). All participants provided written informed consent. Experimental procedures were approved by the local Institutional Review Board and adhered to the Declaration of Helsinki. The participants were monetarily compensated for their time.

Participants were randomly assigned to one of four groups: rTMS/no-go (n = 15), rTMS/neutral (n = 15), sham/no-go (n = 15), or sham/neutral (n = 15). As presented in **Table 2**, participants in each group were similar in BMI, level of subjective hunger reported prior to the taste test, chocolate snack preference, self-reported impulsivity (BIS-11) (20), intention to restrict food intake (RS; 21), and pre- and post-rTMS PANAS mood scores. However, one way ANOVA by age revealed a significant group effect [*F*(3, 56) = 2.81, *p* = 0.047]. Scheffé post-hoc analyses indicated that all groups were similar in age (**Table 2**) but the rTMS/Neutral group was marginally younger than the sham/neutral group (*p* = 0.057).

**Table 2:** Participant characteristics by group in Experiment 2. Means and standard deviations are shown. Statistical results from one-way ANOVAs with a factor of group are presented.

	rTMS/ No-Go	rTMS/ Neutral	Sham/ No-Go	Sham/ Neutral	F(1,56)	*p*
Age	22.93 (3.33)	21.13 (1.60)	23.80 (2.18)	22.33 (2.89)	2.81	0.05
BMI	20.47 (2.30)	21.21 (2.03)	22.03 (3.38)	20.60 (1.94)	1.23	0.31
Hunger	2.27 (0.46)	2.33 (0.49)	2.33 (0.49)	2.00 (0.53)	1.56	0.21
Chocolate snack preference (1–5)	4.40 (0.63)	3.87 (0.99)	3.87 (1.13)	4.40 (0.74)	1.78	0.16
Impulsivity (BIS-11)	52.13 (9.91)	48.00 (10.17)	53.60 (7.78)	50.93 (10.04)	0.93	0.43
Restraint Scale (RS)	15.20 (6.60)	15.40 (5.15)	12.87 (5.58)	13.33 (5.31)	0.77	0.52
PANAS						
Positive—Baseline	21.40 (7.17)	20.00 (4.23)	20.60 (5.22)	24.67 (5.70)	2.01	0.12
Positive—After rTMS	17.27 (5.99)	15.67 (4.34)	18.07 (6.25)	18.53 (8.07)	0.60	0.62
Negative—Baseline	13.00 (2.27)	15.00 (5.15)	13.47 (2.47)	14.33 (4.76)	0.79	0.51
Negative—After rTMS	11.27 (2.22)	11.93 (2.46)	10.53 (1.06)	10.53 (1.55)	1.87	0.15

F is the F-statistics from one-way ANOVA; P is the probability value from one-way ANOVA.

*High-frequency rTMS.* Participants received one session of rTMS using a Magstim Rapid2 (Whitland, UK) equipped with a double 70-mm air film coil. The stimulation site was the left DLPFC, defined as the F3 region of the International 10-20 system (24). Stimulation frequency was set to 10 Hz. Stimulation intensity was fixed at 40% of the maximal stimulator output (MSO). The intensity level was determined by our pre-experimental testing as the level at which no discomfort, such as headache or muscle pain, was reported. The use of MSO as an index of stimulation intensity has been increasingly reported, especially in studies involving stimulation of non-motor cortical regions (25, 26). Previous research indicates that individual resting motor thresholds may not inform the thresholds of other cortical regions (25, 27) and effect sizes in studies using MSO were comparable to those in studies using resting motor threshold as an index of intensity (28).

For the rTMS group, rTMS stimulation was applied in four consecutive blocks. Each block consisted of 10 trains of 3 s that were repeated every 10 s (1200 pulses total). There was a 1 min interval between blocks during which the participants rested. For the sham group, sham stimulation was applied in the same manner except that the coil was placed orthogonally to the skull so that only one edge of the coil rested on the scalp. Mood was assessed using the PANAS (22) before and after rTMS.

*Food-inhibition association training and taste test.* The go/no-go task was used for inhibitory training using the same procedure described in Experiment 1. Briefly, no-go training involved consistent pairings of pictures of chocolate snacks with no-go cues and neutral training involved pairings of pictures of chocolate snacks with both no-go and go cues at equally frequencies. The taste test was carried out using the same procedure as in Experiment 1.

*Procedures.* As in Experiment 1, participants were ostensibly told that they would be participating in two separate studies: one investigating the effect of brain stimulation on motor response and the other evaluating the taste of chocolate snacks. After providing consent, participants proceeded to the stimulation phase and received either rTMS or sham treatment depending on their group assignment (**Figure 1**). Immediately after the stimulation phase, participants performed the go/no-go task. After completion of the go/no-go task, participants reported their current level of subjective hunger on a three-point scale (1: not hungry at all, 2: hungry, 3: very hungry).

Next, participants performed a taste test and evaluated the chocolate snacks for 7 min. The reduction in duration from 10 min in Experiment 1 to 7 min in Experiment 2 was because all participants in Experiment 1 finished the taste test in under 7 min. After the taste test, participants were asked to report if they thought the 2 studies were related and/or if the first study influenced their performance in the second study. Participants were debriefed and thanked for their time. All data analyses were conducted using SPSS 23 (IBM SPSS Statistics, IBM Corp, Armonk, NY, USA).

## Results

### Experiment 1

**Figure 2:**
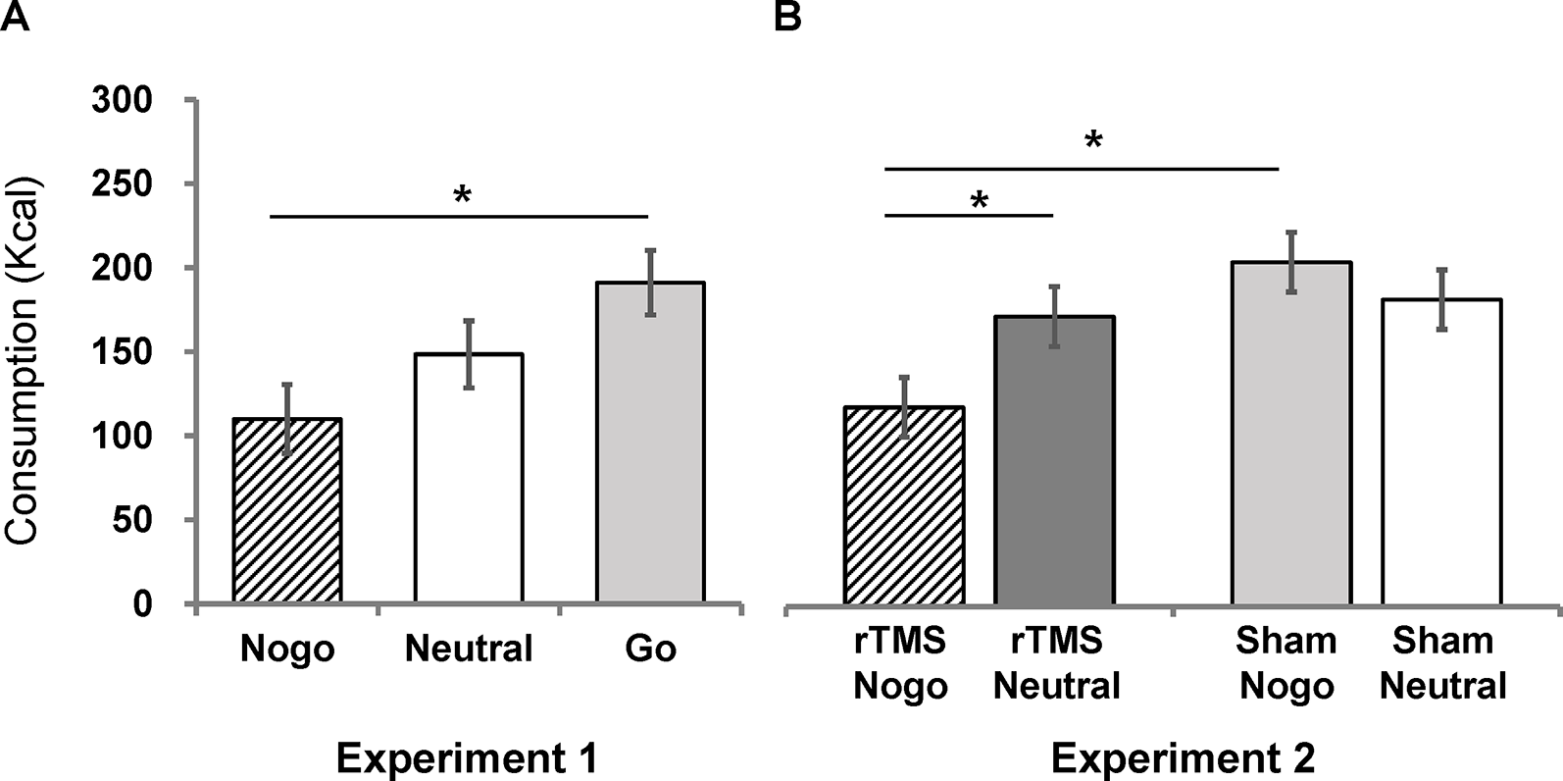
Estimated marginal means (corrected by preference scores and restraint scores) of chocolate snack consumption for each group in Experiment 1 **(A)** and Experiment 2 **(B)**. Error bars indicate standard errors of the mean. An asterisk (*) indicates *p* < 0.05.

Total snack consumption was analyzed using one-way analysis of covariance (ANCOVA), with a factor of group controlling for self-reported snack preference and food restraint scores. We found a significant main effect of group [*F*(2, 37) = 4.18, *p* = 0.02, η^2^ = 0.18]. Post-hoc Bonferroni comparisons of estimated marginal means indicated that the no-go group (M = 109.99, SEM = 20.50) consumed significantly fewer total calories than the go group (M = 191.08, SEM = 19.25; p = 0.019), but not the neutral group (M = 148.44, SEM = 19.98; *p* = 0.56). There was no significant difference between the go group and the neutral group (*p* = 0.41; **Figure 2A**).

### Experiment 2

The effect of combined rTMS and no-go training on chocolate snack consumption was analyzed using a 2 (stim: rTMS vs. sham) × 2 (inhibition: no-go vs. neutral) analysis of covariance (ANCOVA), controlling for self-reported chocolate snack preference and food restraint scores. There was a significant main effect of food-inhibition association training [*F*(1, 54) = 7.65, *p* = 0.01, *η* ^2^ = 0.12] but no main effect of rTMS stimulation [*F*(1, 54) = 0.79, *p* = 0.38]. The interaction between inhibition training and stimulation was significant [*F*(1, 54) = 4.34, *p* = 0.04, *η* ^2^ = 0.07]. Simple effect analyses and post-hoc Bonferroni comparisons of estimated marginal means indicated that the rTMS/no-go group (M = 118.71, SEM = 17.79) consumed significantly fewer calories than the rTMS/neutral group (M = 205.24, SEM = 17.73; **Figure 2B**), *F*(1, 54) = 11.70, *p* = 0.001, *η* ^2^ = 0.18, meaning that the no-go training was effective when combined with rTMS. In contrast, the sham/no-go group (M = 172.75, SEM = 17.87) did not differ from the sham/neutral group [M = 182.91, SEM = 17.69; *F*(1, 54) = 0.16, *p* = 0.69], meaning that the no-go training was not effective after sham stimulation. Further, the rTMS/no-go group consumed significantly less than the sham/no-go group [*F*(1, 54) = 4.41, *p* = .04, *η* ^2^ = 0.08]. Lastly, the rTMS/neutral and sham/neutral groups did not differ [*F*(1, 54) = 0.77, *p* = 0.38].

## Discussion

We conducted two experiments to examine whether the consumption of tempting food is modulated after a combined cognitive and brain stimulation intervention. In Experiment 1, we found that the no-go training group consumed fewer calories of chocolate snacks than the go training group during a taste test. However, no difference was found between the no-go and neutral training groups. In Experiment 2, combined with high-frequency rTMS over the left DLPFC, no-go training effectively reduced the intake of chocolate snacks compared with the effect of neutral training. Consistent with the results of Experiment 1, no-go training with sham stimulation did not have an effect on chocolate snack intake compared with the effect of neutral training combined with sham stimulation. Together, these results suggest that food-inhibition association training combined with high-frequency rTMS may enhance self-control over the intake of tempting food.

Our finding that rTMS/no-go training reduced chocolate snack intake compared with the effect of rTMS/neutral training (as shown in Experiment 2) addresses an ambiguity raised in relation to the interpretation of reduced food craving and/or intake after no-go training versus go training (6). That is, relative reduction of food intake in no-go training in comparison to go training observed in Experiment 1 of this study can be explained by increased disinhibition being associated with go training rather than increased inhibition being associated with no-go training (5, 29). However, as we found in Experiment 2, the reduction of food intake in the rTMS/no-go group compared to the rTMS/neutral training group suggests that indeed inhibitory responses to tempting chocolate snacks can be induced after no-go training. This finding supports the idea that no-go training, in which chocolate-related images were repeatedly and consistently associated with a response inhibition, develops an automatic association between the chocolate snacks and engagement of inhibitory responses (2). This association may be weak following a single session of inhibition training but may become strong enough to produce a significant reduction in food intake with rTMS combined training.

In contrast, the lack of difference in snack intake between rTMS and sham stimulation in the absence of no-go training supports the previous claim that brain stimulation works best for actively engaged neural circuitry (15–17). No-go training engages the dorsolateral prefrontal system related to inhibition of food temptation (18, 19), and this active process likely benefits from rTMS. Without securing such a target process through specific tasks, brain stimulation may risk uncontrolled and random effects. Further studies are warranted to test the specificity of the influences of concurrent brain stimulation and cognitive training tasks on the target process (e.g., controlling craving).

One of the crucial factors to consider in brain stimulation research is the stimulation target (9). The target site for this study was the left DLPFC. Previous brain stimulation studies consistently showed that rTMS over the left DLPFC influenced performance of a go/no-go task (18, 19). Furthermore, the DLPFC is thought to play a modulatory control over appetitive food cravings through functional connections to cortical and subcortical reward processing regions such as the ventromedial prefrontal cortex and ventral striatum (13). Experimental attenuation of the left DLPFC in studies using inhibitory rTMS increased neurocognitive responses to, and consumption of, calorie-dense appetitive foods (30). It is notable, however, that lesion studies and functional magnetic resonance imaging (fMRI) studies have consistently implicated the right ventrolateral prefrontal cortex (VLPFC) in the successful inhibitory control (31–33). A recent electrocorticography study suggested a functional dissociation between the DLPFC and VLPFC in response inhibition (34). Specifically, the DLPFC is involved in the representation of task goals (12), whereas the VLPFC is engaged in the implementation of actual action inhibition as a brake (32, 34). Based on this view, the decreased intake of chocolate snacks following rTMS combined with no-go training can be explained by the facilitated representation of food-inhibition association, an implicitly formed task goal (33). Although we did not examine the effect of VLPFC stimulation in this study, it is worth investigating whether high-frequency rTMS over the right VLPFC produces similar regulatory or dissociable effects as compared with left DLPFC stimulation. Identifying the exact effects of brain stimulation over cortical regions may not only advance our understanding of the modulatory roles of high-frequency rTMS, but also contribute to the development of effective intervention protocols for better clinical outcomes.

Findings from the current study suggest that combining high-frequency rTMS over the left DLPFC and food-inhibition association may reduce consumption of tempting high-calorie foods. The small sample size, which limits our ability to draw stronger conclusions, falls within the range of those described in a recent review of similar studies investigating the effect of brain stimulation on food craving and consumption (35). Despite the small sample size, we replicated the effects of no-go training in the direction of expectation across two experiments, which minimizes the likelihood of spurious findings. Additionally, although we controlled for the influence of individual differences in restraint and snack preference scores in our examination of the effect of training on snack consumption, the possibility of residual confounding cannot be eliminated. An additional limitation is that our participants were healthy young females without overweight and eating disorders. To demonstrate the clinical effects of combined rTMS and no-go training, studies with a larger sample size and involving clinical populations are required. Finally, to identify a definite additive effect of combined rTMS and no-go training as opposed to no-go training alone, a within-subject design would be ideal, in which the same individuals participate in both conditions (rTMS with no-go training and sham treatment with no-go training), with temporal separation. Despite these limitations, we believe that this study will contribute to the generation of more sophisticated hypotheses to further test and implement effective combined cognitive and brain stimulation strategies for developing adaptive eating behaviors.

## Data Availability Statement

All datasets generated for this study are included in the article/supplementary material.

## Ethics Statement

The studies involving human participants were reviewed and approved by Korea University Institutional Review Board. The patients/participants provided their written informed consent to participate in this study.

## Author Contributions

HA: Conceptualization, methodology, investigation, formal analysis, writing–original draft preparation. B-JH: Conceptualization, writing–reviewing and editing. SK: Conceptualization, writing–original draft preparation, reviewing and editing, supervision, funding acquisition.

## Funding

This work was supported by a National Research Foundation of Korea grant funded by the Ministry of Science and ICT (NRF-2017M3C7A1041823) and the Ministry of Education (NRF-2017R1D1A1A09000664), Republic of Korea.

## Conflict of Interest

The authors declare that the research was conducted in the absence of any commercial or financial relationships that could be construed as a potential conflict of interest.
